# Editorial: Neonatal Screening for Congenital Adrenal Hyperplasia in Turkey

**DOI:** 10.4274/jcrpe.galenos.2019.2019.0001

**Published:** 2019-02-20

**Authors:** Abdullah Bereket

**Affiliations:** 1 Marmara University Faculty of Medicine, Department of Pediatrics, Division of Pediatric Endocrinology, İstanbul, Turkey

Newborn screening (NBS) for congenital diseases is among the biggest achievements of modern medicine in the field of public health (1). As a secondary prevention tool, NBS is aimed at early detection of asymptomatic infants affected by certain inborn diseases, an accomplishment which will facilitate early diagnosis, thus with proper treatment, will prevent further complications and sequelae and ensure a better quality of life. Sixty years ago, Guthrie and Susi developed a method that allowed early detection of phenylketonuria, the first disease to be screened in the newborns (2). Later addition of thyroid function tests in the mid 1970s to NBS programs facilitated early detection and treatment of hypothyroidism, thus, largely eliminating the neurodevelopmental impairment from both of these congenital diseases. Since then, screening neonates for dozens of congenital diseases became a routine in most of the developed countries with the objective to initiate early treatment and prevent morbidity and mortality associated with them, depending on the sources for healthcare. Cost-effectivity is one of the important considerations when implementing NBS programs. The diseases screened should be frequently seen in that community, treatable when diagnosed early and result in irreversible sequelae if left untreated.

Turkey still has a high consanguinous marriage rate which results in higher incidence of recessively inherited congenital diseases (3). Nevertheless, implementation of NBS in Turkey was relatively late, the first neonatal screening attempts starting at Hacettepe University in the 1980s for phenylketonuria and sponsored by the Turkish Scientific and Research Centre, followed by hypothyroidism in 1990s (4,5). After these pioneering studies, a countrywide-universal screening program executed by the Ministry of Health finally initiated in 2006 which covered phenylketonuria and hypothyroidism in the beginning with gradual expansion of the program to include screening for biotinidase deficiency starting in 2008 and cystic fibrosis in 2015. The program is highly effective with a current coverage rate of 99% all over the country (6).

Classical congenital adrenal hyperplasia (CAH) is the most common form of of primary adrenal insufficiency in childhood and is a potentially life-threatening condition that requires accurate diagnosis and urgent treatment with glucocorticoid and mineralocorticoid replacement (7). CAH occurs in 1:13,000 to 1:15,000 live births. The most common form of CAH is 21-hydroxylase enzyme deficiency (21-OHD) which constitutes 90 to 95% of all cases of CAH. Approximately 75% of infants with classical 21-OHD have the severe salt-wasting form of the disease that, if not promptly diagnosed and treated, can cause death in early infancy from shock, hyponatremia, and hyperkalemia. While, affected female newborns have ambiguous genitalia, male infants appear normal and and may easily be overlooked.

In 2002 the Joint Lawson Wilkins Paediatric Endocrine Society/European Society for Pediatric Endocrinology Working Group recommended biochemical screening for CAH in the newborn period. Neonatal screening for CAH is effective in detecting the salt-wasting form and thereby reducing mortality (8).

In this issue of JCRPE, Güran et al (9) present the first pilot study of NBS for 21-OHD in Turkey in nearly 40,000 neonates to estimate incidence of CAH in Turkey and to assess the characteristics and efficacy of the adopted newborn CAH screening strategy. The study was carried out under the authority of the Turkish Directorate of Public Health, currently the main organisation responsible for nationwide NBS programs. The screening protocol included one sample two-tier testing with measurement of 17α- hydroxyprogesterone (17-OHP) the most abundant substrate for the CYP21 enzyme by fluoroimmunoassay in the first step in dried blood spots obtained on the 3-5^th^ days of life followed by steroid profiling in the same dried blood spots using liquid chromatography-tandem mass spectrometry (LC-MS) method to measure 17-OHP, 21-deoxycortisol, cortisol, 11-deoxycortisol and androstenedione as a second-tier test in those with positive initial screening. The babies with a steroid ratio (21-deoxycortisol+17-OHP)/cortisol of ≥0.5 were referred to pediatric endocrinology clinics for further diagnostic assessment. They found that of the 38,935 infants tested, 2265 (5.82%) had second-tier testing, and 212 (0.54%) were referred for clinical assessment. Final analyses showed that six cases of CAH were picked up (four males, two females). Four cases were identified as salt-wasting 21-OHD (two males, two females), one male baby had simple virilizing 21-OHD, and one male baby had 11-OHD CAH. The incidence of classical 21-OHD in the screened population was 1:7,787.

Incidence ratio obtained in this study is among the highest detected so far, confirming the necessity of including 21-OHD screening in the extended NBS program in Turkey. This high incidence is mostly due to the high rate of consanguineous marriages in Turkey (overall 22%, increasing to 34% in South East Anatolia region) giving rise to higher incidence of homozygous biallelic mutations in this population together with compound heterozygotes for two or more different mutant *CYP21A2* alleles (3). Not surprisingly, 3 of 5 patients identified in the current study were homozygous carriers of biallelic mutations causing classical 21-OHD. Furthermore, the high carrier rate for classical CAH in the general population is another reason for this relatively higher incidence of classical CAH in Turkey. Finally, the high efficiency of the screening strategy owing to relatively lower cut offs, hence not missing any case, might have also contributed to the high incidence obtained in the study.

At present, NBS for 21-OHD is carried out in more than 50 countries worldwide. The majority of screening programs use a single screening test which might result in some cases to go undiagnosed even with screening (10). To improve the efficacy of screening, some screening programs reevaluate samples with borderline first-tier test results with a second-tier test and some implement repeat screening in this situation. Because of the high false-positive rate of immunoassay methods, LC-MS was recommended as a second-tier test (11). Mass spectrometry is an analytical technique that allows the identification and quantification of compounds in a biological sample according to the mass/charge ratio. This methodology makes possible the simultaneous measurement of several metabolites and, consequently, ratio measurements of one analyte in respect to another are also possible, which improves the specificity of the screening. In 21-OHD screening, as done in this study, some programs measure the concentration of different hormones (17-OHP, 21-deoxycortisol, and cortisol) as a second-tier test on samples with a positive first-tier test result (12). Some US states mandate organic solvent extraction prior to immunoassay of dried blood spots in order to increase specificity. A search for the best screening approach which detects patients earlier without increasing false negative cases is an ongoing challenge in this area.

Single sample, two-tier screening strategy as used in this study, carries some risk in missing CAH cases with delayed rise in 17-OHP, but obtaining a second sample at a later time would further complicate the screening and cause further delay in obtaining results. As in all screened diseases, a high level of suspicion in patients presenting with signs and symptoms of CAH should still be maintained even if the baby has false-negative screening results. Furthermore, meticulous search for any adrenal-related crises/deaths in the screened area/population is important for evaluation of the efficacy of screening strategy, an activity which requires reliable hospital and/or registry records.

Ideally, screening results should be available before approximately two weeks of age, when salt-wasting typically becomes evident. However, in the current study, the initiation of hydrocortisone treatment ranged between 10 to 30 days of life in 4 cases with salt-wasting 21-OHD and the duration from birth to clinical evaluation of abnormal screening test results of false positive cases was 25.8±6.4 days, results which indicate a delay. Fortunately, none of the patients (except one with hyponatremia) were in critical condition despite this delay. The authors rightly identified the reasons for this delay and suggested possible solutions (accelerating sample transport times, performing both steps of screening in a single central laboratory so that second-tier testing can be performed on the same day upon positive first screening, defining an alarm level of 17-OHP in the first tier and sending newborns with 17-OHP above this level directly for clinical evaluation without waiting for second tier results etc.) to optimize screening program, which is the main objective of doing pilot studies.

For any screened disease, recall rate should be in a reasonable range since referral of these babies to clinics for further confirmation of the diagnosis and initiation of treatment require considerable work, time and cost. This is particularly important for countries where the healthcare system is overloaded and the sources are limited. In addition, parents of infants with positive screens may suffer significant psychological distress until the final diagnosis is reached. In that respect, recall rate was higher (5.82% in the first and 0.54% in the second tier testing) despite two-tier screening approach in this pilot study compared to previous studies which reported a figure between 0.002% and 1.2% (13). This is due to lower cut-off values the authors used for the first step 17-OHP FIA measurements as well as the second step (21-S+17-OHP)/F ratio to increase the sensitivity of this pilot study. However, based on the obtained results, elevating cutoff values two-fold would seem to reduce false positives dramatically without decreasing sensitivity. (taking 20 instead of 10 ng/mL for the first tier 17-OHP and taking (21-S+17-OHP)/F ratio ≥1 instead of ≥0.5) thus potentially eliminating this problem in the future extended NBS program in Turkey. This observation is in line with that reprted by Janzen et al (12) who found that none of the cases with CAH had a (21-S+17-OHP)/F ratio <1. It appears that using the above-mentioned cut-off values may enable less labour intensive, and more efficient screening strategy for CAH with a better cost-benefit profile.

Thanks to the LC-MS method, another “bonus” of this pilot study was diagnosing a male newborn with classical 11 hydroxylase deficency, who might be the first patient with 11-OHD identified directly during NBS for CAH. Second-tier LC-MS also measures 11-S in addition to 17-OHP, 21-S, cortisol and 4AS, which is specifically diagnostic for 11-hydroxylase deficiency. Thus, the method of steroid profiling has a potential to distinguish other rare forms of classical CAH, beyond 21-hydroxylase deficiency. Even though the hormones measured in LC-MS/MS based panels are not specifically diagnostic for the rare forms of CAH, perturbations in simultaneous steroid measurements have the potential to provide preliminary information suggesting the need for further evaluation. This is particularly important for countries like Turkey where “rare” forms of classic CAH are common due to high rate of consanguinity.

Analysis of the data from the current study is a big step towards optimization of the 21-OHD screening in Turkey. For no doubt, the incidence of 21-OHD obtained in this study calls for inclusion of 21-OHD screening in the NBS panel in Turkey. It would be interesting to see the figures after the implementation of countrywide screening in the years to come.
